# Enhanced Photoelectrochemical Detection of Bioaffinity Reactions by Vertically Oriented Au Nanobranches Complexed with a Biotinylated Polythiophene Derivative

**DOI:** 10.3390/s90201094

**Published:** 2009-02-19

**Authors:** Huiqiong Zhou, Yanli Tang, Jin Zhai, Shu Wang, Zhiyong Tang, Lei Jiang

**Affiliations:** 1 National Center for Nanoscience and Technology, Beijing 100190, P.R. China; 2 Key Laboratory of Organic Solids, Institute of Chemistry, Chinese Academy of Sciences, Beijing 100190, P.R. China

**Keywords:** Nanostructure, Photoelectric conversion, Photoelectrochemical biosensor

## Abstract

Four nanostructured Au electrodes were prepared by a simple and templateless electrochemical deposition technique. After complexing with a biotinylated polythiophene derivative (PTBL), photocurrent generation and performance of PTBL/Au-nanostructured electrodes as photoelectrochemical biosensors were investigated. Among these four nanostructured Au electrodes, vertically oriented nanobranches on the electrode significantly improved the photoelectric conversion, because the vertically oriented nanostructures not only benefit light harvesting but also the transfer of the photogenerated charge carriers. Owing to this advantaged nanostructure, the PTBL/Au-nanobranch electrode showed higher sensitivity and faster response times in the photoelectrochemical detection of a streptavidin-biotin affinity reaction compared to a PTBL/Au-nanoparticle electrode.

## 1. Introduction

The principle of photoelectrochemistry has been utilized extensively in construction of photoelectric devices dealing with energy transfer and conversion [1-3], but the universally low photoelectric conversion efficiency hinders practical applications of this type of devices. Many researchers have explored the introduction of nanostructures into photoelectric devices to improve their conversion efficiency [4-8]. Nanoparticles [9-12], porous materials [13-16], and micro/nano-scale nanostructrues [17-18] have been used to construct the electrodes of photoelectric devices, which were successfully shown to display improved performance. Recently, several works have shown that vertically oriented nanostructures exceptionally benefited the transfer of photogenerated charge carriers, and further magnified photocurrents in the circuits remarkably [18-19]. However, current methods of fabrication of vertically oriented nanostructures, such as the hydrothermal method [18-19] or templated synthesis [20-21] are time-consuming and laborious.

As one type of promising and inexpensive photoelectric devices, photoelectrochemical biosensors are an alternative to conventional analytical methods due to their high sensitivity and potential in array analysis. In this method, conductive polymers [22], transition metal complexes [23-26], semiconductor nanoparticles [27-31] or other semiconductor nanostructures [32] are widely used as photosensitizers on conducting electrodes. The electrons of the photosensitizers are excited from their ground state to the excited state to produce electron-hole pairs after absorbing photon energy. If an electrode with an appropriate energy level close to either the conductance band or valence band of the photosensitizers is used, the photoexcited electrons or holes transfer to the electrode and produce a photocurrent. In this process, a biosensor will be implemented if the detected biomolecules can specifically change the amplitude of the produced photocurrent. Evidently, any enhancement of the photoelectric conversion efficiency should improve the performance of a photoelectrochemical sensor.

Herein, a simple and templateless electrochemical method was used to fabricate nanostructured Au electrodes with the aim of enhancing the photoelectric conversion of a biotinylated polythiophene derivative (PTBL). The PTBL molecule, which consists of both polythiophene backbones with high photoelectrical response and biotin branches with specific biological recognition, was used as a photoelectrochemical sensitizer. Au nanostructures were employed to construct the conductive electrodes because Au is one of the most common electrode materials that is both stable and biocompatible, and its nanostructures are easily fabricated and controlled. As expected, the photocurrent generated by PTBL was amplified by these nanostructured Au electrodes, particularly by a vertically oriented nanobranch surface. The use of the PTBL/Au-nanobranch electrode as a novel photoelectrochemical biosensor was explored, and it showed a remarkable enhancement in response to streptavidin binding when the photocurrent change of the system was monitored, compared to a PTBL/Au-nanoparticle electrode.

## 2. Results and Discussion

### 2.1. Characterization of nanostructured Au electrodes

**Figure 1.**
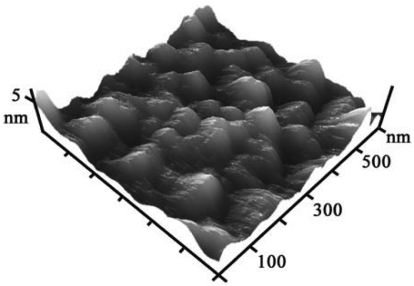
AFM image of Au nanoparticle surface formed by sputtering. The diameters of the nanoparticles were around 3 nm.

**Figure 2.**
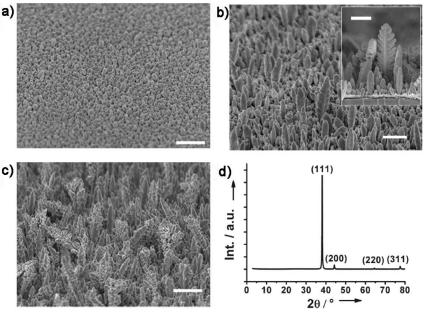
SEM images of nanostructured Au electrodes by electrochemical deposition at -0.2 V for a) 400, b) 800, c) 1200 s; The inset in b) is side view from 90°, and other SEM images are side views from 45°, all scale bars are 500 nm; d) XRD patterns of Au nanobranch electrode fabricated at -0.2 V for 800 s.

Templateless electrochemical deposition was used to fabricate nanostructures on the Au nanoparticle surfaces. In a typical procedure, an Au nanoparticle layer was sputtered on the substrate to produce nucleation sites for further electrochemical deposition. The diameters of the nanoparticles were around 3 nm, and they were closely packed to form a rather uniform layer (Figure 1). After a short deposition time of 400 s at -0.2 V, prominent particles appeared (Figure 2a).

**Figure 3.**
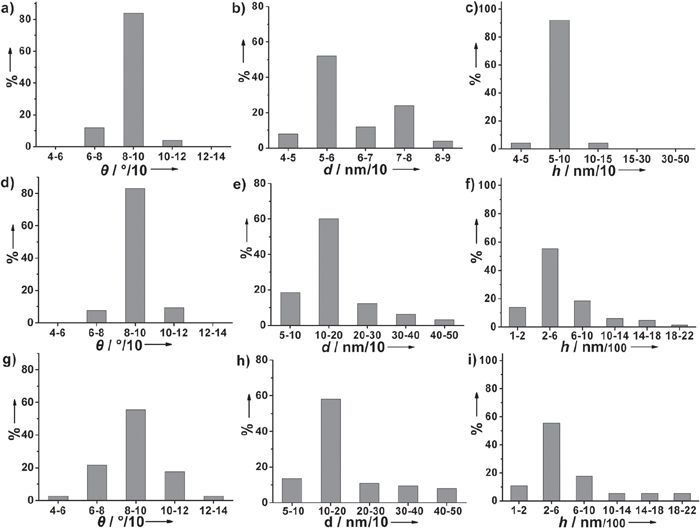
Distribution of angles, diameters and heights of Au nanostructures electrodeposited at -0.2 V for different time intervals: (a, b, c) 400 s, (d, e, f) 800 s, (g, h, i) 1200 s.

When the time was extended to 800 s, the particles grew into the separated and vertical Au nanobranch structures. The diameters and heights of the nanobranches were 50-500 nm and 100-2,000 nm, respectively, and more than 80% nanobranches stood vertically to within 10° of the substrate normal (Figure 2b and Figure 3). The nearly epitaxial joints of stem and branch (inset in Figure 2b) had a fixed angle of around 45°. When the electrodeposition time was prolonged to 1,200 s (Figure 2c), most nanobranches grew into fractal nanocorns. The protruded and coarse Au nanocorns with faster growth speed bent toward the substrate surface, and thus resulted in broadening in the angle distribution vertical to within 40° of the substrate normal (Figure 3).

**Figure 4.**
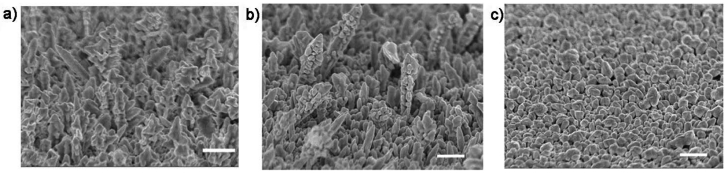
SEM images of nanostructured Au electrodes by electrochemical deposition at **a)** -0.1, **b)** -0.3, **c)** -0.4 V for 800 s. All SEM images are side view from 45°, all scale bars are 500 nm.

**Figure 5.**
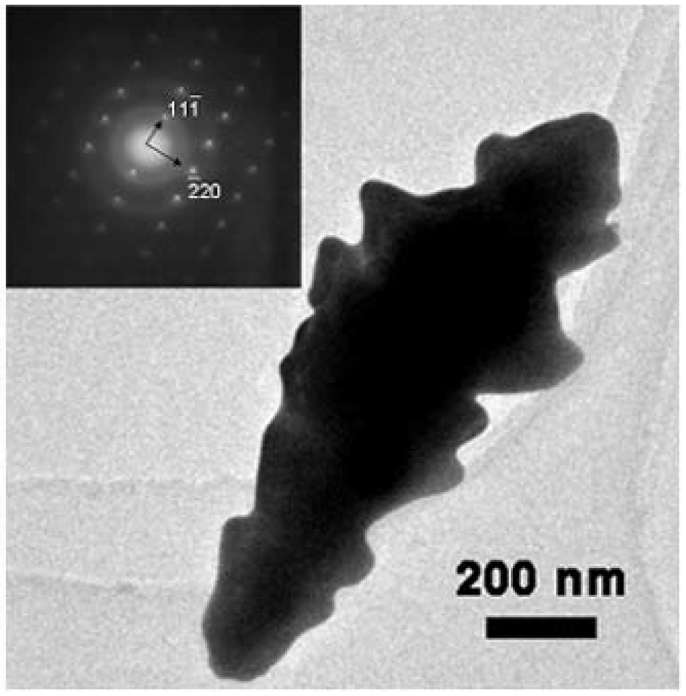
TEM image of a single Au nanobranch fabricated at -0.2 V for 800 s. The inset is the selected area diffraction pattern taken from [112] direction of Au nanobranch.

The loss of orientation was consistent with the recent observation that many Au microclusters were randomly formed on the surface after electrodeposition for 45 minutes [33]. The morphology of the deposited nanostructured Au electrodes also depended on the deposition potential. At the time interval of 800 s deposition potentials of -0.1 V (Figure 4a) and -0.3 V (Figure 4b) led to nanocorn structures with poor vertical orientation, while a deposition potential of -0.4 V resulted in a nanopebble structure (Figure 4c). Altogether, a deposition potential of -0.2 V and a deposition duration of 800 s are superior conditions for the preparation of vertically oriented Au nanobranches. The nanostructure evolution of Au electrodes follows a typical electrocrystallization process of metal on the surfaces, and the formation of the branched nanostructures should arise from the secondary nucleation at the front face of the newly formed metals in diffusion-controlled growth [34-35]. The absolutely stronger diffraction peak of Au (111) relative to the (200), (220), and (311) ones in the X-ray diffraction (XRD) patterns reveals that the Au nanobranches oriented on the substrates have a preferential growth along the [111] direction (Figure 2d). The same conclusion is drawn from selected area electron diffraction (SAED) in transmission electron microscopy (TEM) (Figure 5), in which the individual Au nanobranch is discerned as a single crystal with a growth direction along the [111] direction. The single-crystalline nature should benefit the transport of charge carriers along Au nanobranches, and thus improve the conversion efficiency in photoelectric processes.

### 2.2. Photocurrent Enhancement

**Figure 6.**
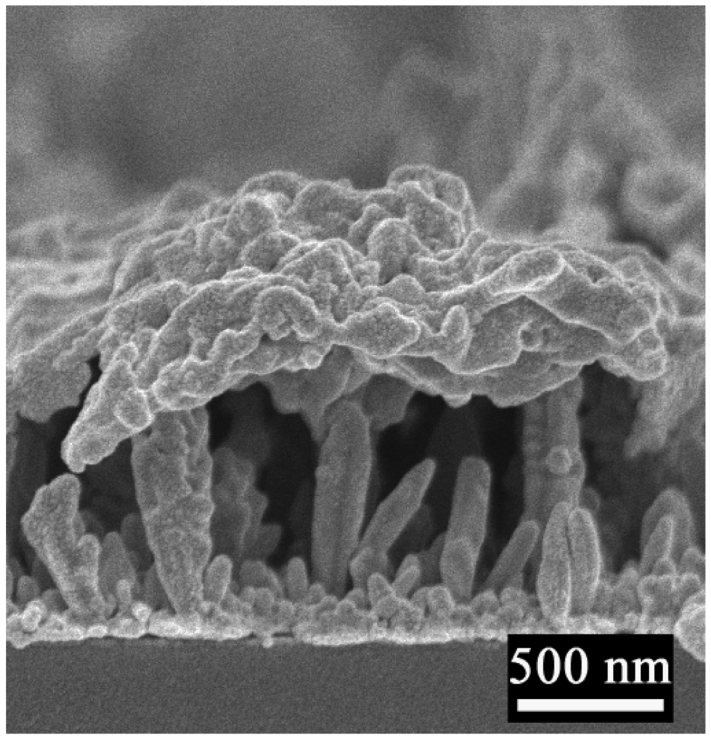
SEM image of PTBL cast on Au nanobranch surface, which prepared by electrochemical deposition at -0.2 V for 800 s.

**Figure 7.**
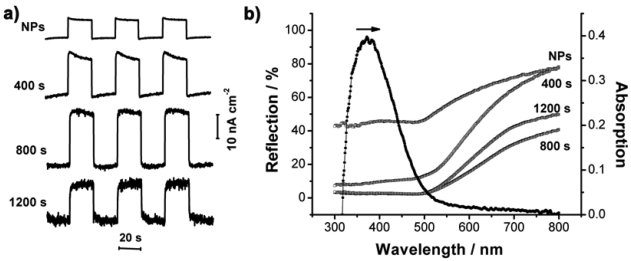
a) Photocurrent of PTBL/Au-nanostructured electrodes in PBS buffer solution by irradiation with 110 mW cm^-2^ of white light at a bias of 0 V; b) reflection spectra of four nanostructured Au electrodes and absorption spectrum of PTBL in DMF solvent; curves NPs, 400 s, 800 s, 1200 s represent the nanostructured Au electrodes prepared by deposition at -0.2 V for 0, 400, 800, and 1,200 s respectively.

Polythiophene polymers are widely applied in photoelectric devices for their high mobility and fast photoelectrical response [36]. One type of polythiophene derivative, PTBL (for its synthesis, see the Experimental section) displayed desirable photoelectric performance in this experiment. A similar quantity of PTBL molecules was cast onto different nanostructured Au electrodes for comparison, and the thickness of the PTBL film was about 200 nm, with relatively good homogeneity (Figure 6). The photoelectric responses of PTBL generated under light irradiation were amplified to different degrees by the different Au nanostructures (Figure 7a). The PTBL/Au-nanobranch electrode (800 s of electrodeposition) showed the largest photocurrent of 24.72 nA cm^-2^, which was nearly three-times larger than that of the PTBL/Au-nanoparticle electrode (NPs).

The optical properties of nanostructured electrodes and the transfer routes of charge carriers are two possible factors that may give rise to the enlargement of the photocurrent in a photoelectric conversion system. First, the Au nanobranch electrodes showed the lowest reflectance over the whole wavelength spanning from 300 to 800 nm in the reflectance spectra (Figure 7b). This favored light harvest for photocurrent generation by the PTBL film. Second, the Au nanobranches (Figure 2b) were separated and oriented, which provided pathways to direct the flow of photogenerated charge carriers from the PTBL film to the electrode's surface. This charge carrier transfer route prevents possible recombination of electron and hole pairs [18]. Similarly to previous reports [15, 37], the total photocurrent yield (*η*) in photoelectric process can be expressed by Equation (1):

(1) $$\eta =\eta_{g}\eta_{d}\eta_{c}$$

where *η_g_* is the quantum efficiency for exciton generation, *η_d_* is the efficiency of exciton dissociation resulting in the generation of carriers, and *η_c_* is the carrier collection efficiency in the external circuit. The creation of excitons originates from the light harvest, and thus a high light harvest efficiency can raise *η_g_*. Photogenerated excitons may either dissociate into electrons and holes at interfaces or undergo recombination in the bulk material. The vertically oriented nanobranched Au electrodes offer larger specific surface area, create more PTBL/electrode or PTBL/electrolyte interfaces, and provide straight routes to transfer the excitons, which is beneficial for charge separation and prevention of recombination, so that *η_d_* is enlarged. Since the vertically oriented nanostructure improves both *η_g_* and *η_d_*, the total photocurrent generated is promoted.

**Figure 8.**
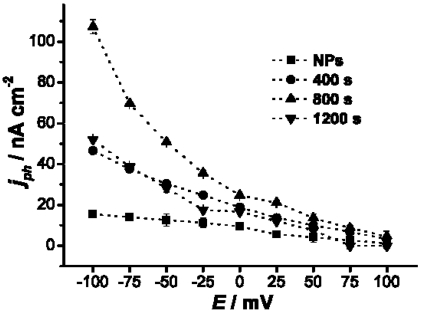
Dependence of bias potential (*E*) on photocurrent (*j_ph_*) of the PTBL/Au-nanostructured electrodes, curves NPs, 400 s, 800 s, 1200 s represent the nanostructured Au electrodes prepared by deposition at -0.2 V for 0, 400, 800, and 1200 s respectively.

**Figure 9.**
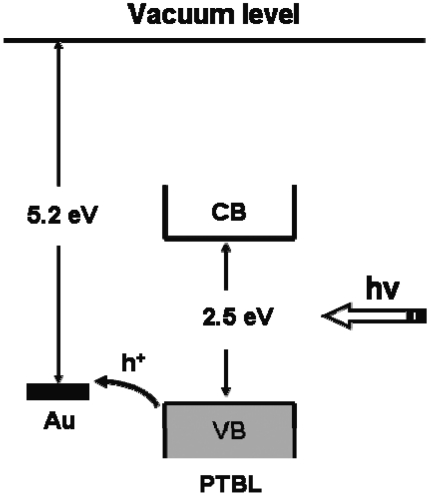
Energy level diagram of PTBL/Au. The band gap of PTBL was evaluated to be 2.5 eV from the absorption edge of the absorption peak of PTBL in figure 7b. The relative positions of the levels of PTBL and Au were adopted from reference [38].

For all of PTBL/Au-nanostructure electrodes, the response of current to on/off cycling of light was quick and reproducible, and the photocurrent remained stable, even after 1,000 cycles. It was noted that nanostructured Au electrodes without a PTBL film coating showed negligible photocurrents, which indicated that the current resulted from separation of electrons and holes of the PTBL films under photoillumination. The relationships between photocurrent and bias potential of PTBL/Au-nanostructure electrodes were also studied (Figure 8). When a negative potential was applied to the electrodes, the electric field promoted the hole transfer from the PTBL film to the Au electrode surface, facilitated the separation of the photogenerated electrons and holes, and led to an increase of the photocurrent. Alternatively, the positive potential suppressed hole transfer, so the cathodic photocurrent was reduced. The hole transfer characteristic is consistent with the energy level diagram, in which the Fermi level of the Au metal is aligned with the valence band of the PTBL molecules and the holes easily jump from the valence band of PTBL to the Au Fermi level (Figure 9).

### 2.3. Photoelectrochemical detection of bioaffinity reaction

**Figure 10.**
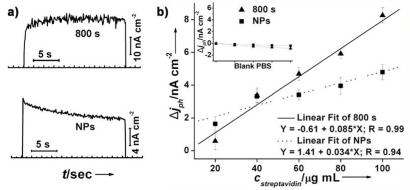
a) Photocurrent response of PTBL/Au-nanobranch electrode (top, deposition at -0.2 V 800 s) and PTBL/Au-nanoparticle electrode (bottom) after binding of 60 μg/mL streptavidin in PBS solution; b) The dependence of streptavidin concentration (*C_streptavidin_*) on photocurrent decrease (*Δj_ph_*) of PTBL/Au-nanobranch electrode (solid line) and PTBL/Au-nanoparticle electrode (dotted line); The inset in b) shows the results of control experiments using blank PBS solution.

The PTBL/Au-nanobranch electrode was used for label-free photoelectrochemical detection of streptavidin binding, and the PTBL/Au-nanoparicle electrode was tested for comparison. It was noted that the photocurrent of the PTBL film was produced without any oxidative quencher but O_2_ in the electrolyte, which provided gentle conditions and benefited biomolecule recognition, while many other systems often need additional an oxidative quencher to maintain the photoelectrochemical cycle [23, 25-26]. Binding of streptavidin didn't influence the photocurrent response speed of the PTBL/Au-nanobranch electrode and no obvious hysteresis is seen in Figure 10a (top line). Correspondingly, the stabilization time of PTBL/Au-nanoparicle electrode in Figure 10a (bottom line) was about 6 s after binding with streptavidin. The quick photoelectrical response should arise from the structure of electrodes in which the Au nanobanches easily realized fast transfer of electrons and holes under photoillumination.

The decreases of photocurrent of the PTBL/Au-nanobranch and the PTBL/Au-nanoparticle electrodes were plotted against different concentrations of streptavidin, and nearly linear dependences were obtained for both (Figure 10b). The slope of the photocurrent decreases against the concentration of streptavidin is 0.085 for the PTBL/Au-nanobranch electrode, which is 2.5 times larger than that of the PTBL/Au-nanoparticle electrode, so we conclude that the PTBL/Au-nanobranch electrode showed high sensitivity towards streptavidin. The decrease in photocurrent after binding with streptavidin was ascribed to steric hindrances in the diffusion of charge carriers of PTBL film to the electrolyte [23, 26]. We also noted that the signal to noise ratio (S/N) of the PTBL/Au-nanobranch electrode was 1.6-fold better, compared with the PTBL/Au-nanoparticle electrode. Though S/N was slightly decreased, the sensitivity (2.5 times) was considerably improved. Therefore, the vertically-oriented structure benefited the PTBL/Au-nanobranch electrode allowing both fast and sensitive response characteristics for biodetection. The detection limit for streptavidin for this type of biosensor is about 5 μg/mL. Control experiments carried out with PTBL/Au electrodes incubated with pure PBS buffer solution showed no decrease in photocurrent intensity (the inset in Figure 10b). This demonstrated that the photocurrent decrease resulted from the specific binding of streptavidin with the PTBL film on electrode surfaces.

## 3. Experimental Section

### 3.1. Synthesis of the PTBL polymer

**Scheme 1.**
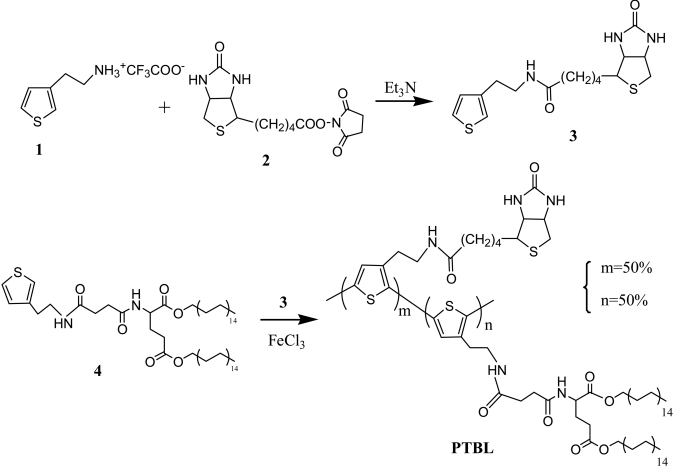
Synthesis of PTBL polymer

The polymer was synthesized by condensation of 3-(4′-biotinbutylacylamine)ethyl thiophene (3) with compound 4 in the presence of FeCl_3_, as outlined in Scheme 1.

#### 3-(4′-Biotinbutylacylamine)ethylthiophene(**3**)

d-Biotin (366 mg, 1.5 mmol), *N*-hydroxysuccinimide (224 mg, 1.95 mmol) and dicyclohexylcarbodiimide (463 mg, 2.25 mmol) were dissolved in dried DMF (12 mL) and stirred at room temperature for two days to give compound **2**. Then a solution of compound **1** (295 mg, 1.3 mmol, previously treated with triethylamine) dissolved in DMF (2 mL) and triethylamine (0.5 mL) were added, and the resulting mixture was stirred for two days. After the reaction was complete, the solvent was evaporated under reduced pressure. CHCl_2_ was added, and the resulting white residue was removed by filtration. The flocculent precipitate was dried under vacuum to provide product **3** (171 mg, 32%). ^1^H-NMR (400 MHz, CDCl_3_): δ = 7.28 (d, *J* = 4.0 Hz, 1 H), 7.01 (s, 1 H), 6.92 (d, *J* = 4.4 Hz, 1 H), 5.81 (s, 1 H, NH), 5.49 (s, 1 H, NH), 4.78 (s, 1H, NH), 4.50 (m, 1H), 4.32 (m, 1 H), 3.51 (t, *J* = 6.4 Hz, 2 H), 3.15 (m, 1 H), 2.94 (m, 1 H), 2.85 (t, *J* = 6.8 Hz, 2 H), 2.73 (m, 1 H), 2.15 (m, 1 H), 1.71-1.29 (m, 6 H); ^13^C-NMR (100 MHz, CDCl_3_): δ = 172.9, 164.7, 156.7, 139.3, 128.1, 125.9, 121.3, 61.7, 60.1, 55.3, 40.5, 39.8, 36.5, 30.1, 28.0, 25.6; MS (EI): 353 (M^+^).

#### Polythiophene copolymer containing biotin and lipid moieties (**PTBL**, **5**)

A solution of compound **4** (40 mg, 0.05 mmol) and compound **3** (35 mg, 0.05 mmol) dissolved in CHCl_3_ (5 mL) were added dropwise to a flask containing CHCl_3_ (10 mL)and anhydrous iron (III) chloride (32 mg, 0.2 mmol) under protection of nitrogen, and then stirred at room temperature for two days. Subsequently, methanol was used to stop the reaction, and the solution of the resulting mixture was removed under reduced pressure. The residue was dissolved in CHCl_3_, precipitated by a copious amount of methanol, this was repeated twice, and the residue then dried to provide PTBL (38 mg, 66 %) as a red solid. ^1^H-NMR (400 MHz, DMSO-d_6_): 8.19 (br), 7.89 (br), 7.42 (br), 7.15 (br), 6.96 (br), 6.39 (br), 4.25 (m), 3.96 (m), 3.60-3.95 (br), 2.9-2.25 (br), 2.03 (m), 1.52 (m), 1.21 (m), 0.83 (s); GPC: *M*_n_ = 87900, *M*_w_ = 126400, PDI = 1.43.

### 3.2. Characterization of nanostructured Au electrodes

After thorough cleaning by piranha solution and water, the glass substrate was precoated by sputtering a Cr layer with a thickness of 0.5 nm, followed by a layer of Au nanoparticles, provided by a SCD 500 sputter coater (Balzers). Subsequently, Au nanostructures were prepared on the surfaces of Au nanoparticles in a mixture solution of 1 mg/mL HAuCl_4_ and 0.1 M H_2_SO_4_ with varying potential levels and durations, using a conventional three electrodes configuration on a CHI630A electrochemical analyzer (Chenhua Instruments Co., Shanghai). The D/max-2500 X-ray diffractometer (Rigaku) with Cu K*α* radiation and the Tecnai G^2^ 20 (FEI) transmission electron microscopy were applied to identify the crystal structure of Au nanostructures. The surface morphologies were attained using a JSM-6700F field emission electron microscope (JEOL) and an Agilent 5500 atomic force microscope. The reflection spectra and absorption spectrum were measured by a U-3010 spectrophotometer (Hitachi).

### 3.3. Characterization of nanostructured Au electrodes

100 μL of 0.25 mg/mL PTBL in DMF solution were drop-cast onto the surface of nanostructured Au electrode (15×15 mm), and naturally dried under ambient conditions. The nanostructured Au electrodes modified with PTBL were immersed in 0.1 M PBS solution in a conventional three-electrode cell with quartz window for photocurrent measurement. The irradiation source was a CMH-250 solar simulator (Aodite Photoelectronic Technology Ltd, Beijing) with a white light of 110 mW cm^-2^.

### 3.4. Characterization of nanostructured Au electrodes

A blocking solution, composed of 5 % (w/v) bovine serum albumin (BSA) in 0.1 M PBS was dropped on the PTBL/Au-nanobranch electrode and left for 2 h at 4 °C to prevent nonspecific binding of streptavidin onto the electrode surface. The electrodes were then carefully washed several times with PBS buffer. Subsequently, 50 μL of PBS solution containing streptavidin with different concentrations were then incubated for 30 min with the biotinylated films. After rinsing with PBS, photocurrent was measured under bias voltage of –75 mV. In the control experiment, the above procedure was repeated except for using blank PBS buffer solution in place of the streptavidin solution.

## 4. Conclusions

In conclusion, fast response times and high sensitivity in the photoeletrochemical detection of the streptavidin-biotin affinity reaction were obtained by introducing vertically oriented Au nanobranches onto electrodes after complexing with PTBL. The vertically oriented Au nanobranches were fabricated by an easy templateless electrochemical deposition method. Even though the morphology of the obtained Au nanobranches was not perfectly homogenous, they were confirmed to increase photocurrent successfully for favouring not only light harvest, but also transfer of the photogenerated charge carriers. This simple strategy remarkably enhanced photoelectric conversion, and may provide insights for the construction of advanced photoelectric devices and other high performance nanostructured biosensors.
